# Supplementary material for: Computational fluid dynamic analysis of the nasal respiratory function before and after postero-superior repositioning of the maxilla
Source: PLoS One. 2022 Apr 28;17(4):e0267677. doi: 10.1371/journal.pone.0267677 (PMC9049540; doi:10.1371/journal.pone.0267677)
Supplement: Attachment — Submitted filename: review.docx [file pone.0267677.s001.docx]

The manuscript offers good insights into the effect of changes in airway morphology due to the repositioning of bone fragments which is beneficial in predicting the effect of such operation on patients ventilation, but the number of cases used in the study is quite small which makes it quite hard to get more generalized conclusions. However, the study approach is quite promising for the future of corrective surgeries.

-          In the abstract, page (2) line (32-33),” the rate of change in the cross-sectional area of the mass extending from the nasopharynx to oropharynx approximated 1”. It is not clear how the rate of change in the cross-sectional area was calculated?

-          In the second paragraph in the introduction, page (4) line (55-69), this paragraph is a bit confusing to the reader, as it is not clear what the authors are trying to address in this paragraph and how it is related to the current work.

-          Figure (1) need to be more intuitive by adding a color legend to address reactionary counter-clockwise rotation during postero-superior repositioning.

-          typo error line (90) page (6),” Surgical impaction of the maxilla and the reaction of the mandible ae illustrated schematically”.

-          It has to be mentioned clearly that the study was performed on females only because the authors mentioned that the study was performed on 3 females.( page 7, line 100).

-          Figure illustrating the mesh is needed to provide information regarding this information “Three layers of the tetrahedral/hybrid tetrahedral-prism”, mentioned in line 129, page(8).

-          Information about the element size and mesh independence study need to be added.

-          Reason for choosing these assumptions is steady and need to be clarified in the text. Line (135), page (9).

-          In page (10) line (162-163), the Outlet boundary condition is used a free outlet and p=0 in one of the cases, can you clarify the reason behind using different outlet boundary conditions.

-          Figure (6), a y-axis label need to be added and the x-axis.

-          Can you indicate how the STENOSIS -1mm, -3mm is measured in the figure (3), it seems to be a distance, so can you clarify how this is measured

-          In page (25), line (327), the assumption of rigid upper airways is quite obsolete; as some studies have investigated the effect of upper airways tissue motion (The effects of upper airway tissue motion on airflow dynamics). Also, most of the experimental studies use flexible materials for manufacturing upper airway replicas’.

-          Body mass index (BMI) for the cases tested in this study needs to be mentioned.
