# Supplementary material for: Computational fluid dynamic analysis of the nasal respiratory function before and after postero-superior repositioning of the maxilla
Source: PLoS One. 2022 Apr 28;17(4):e0267677. doi: 10.1371/journal.pone.0267677 (PMC9049540; doi:10.1371/journal.pone.0267677)
Supplement: Attachment — Submitted filename: Response to Reviewers.doc [file pone.0267677.s003.doc]

Shaokoon Cheng
Academic Editor
*PLOS ONE*

Dear Dr. Cheng:

Subject: Submission of revised paper PONE-D-21-36984R1

Thank you for giving me the opportunity to submit a revised draft of my manuscript titled “Computational fluid dynamic analysis of the nasal respiratory function before and after postero-superior repositioning of the maxilla” to *PLOS ONE*.

We appreciate the time and effort that you and the reviewers have dedicated to providing your valuable feedback on our manuscript. We have revised the manuscript according to the comments from editors and reviewers.

We have highlighted the changes within the manuscript.

Here is a point-by-point response to the journal requirements and the reviewers’ comments.

Journal Requirements

*Please review your reference list to ensure that it is complete and correct. If you have cited papers that have been retracted, please include the rationale for doing so in the manuscript text, or remove these references and replace them with relevant current references. Any changes to the reference list should be mentioned in the rebuttal letter that accompanies your revised manuscript. If you need to cite a retracted article, indicate the article’s retracted status in the References list and also include a citation and full reference for the retraction notice.*

**Reply:**

We have ensured that our reference list is complete and correct. Additional reference lists have noted as follows:

20.	Hoppenreijs TJ, Stoelinga PJ, Grace KL, Robben CM. Long-term evaluation of patients with progressive condylar resorption following orthognathic surgery. Int J Oral Maxillofac Surg. 1999;28: 411-418. doi: [10.1034/j.1399-0020.1999.280602.x](https://doi.org/10.1034/j.1399-0020.1999.280602.x).

Comments from Reviewer #1

*The manuscript is significantly improved.*

*However, the atmospheric pressure mentioned in page 10 line 152 is still inaccurate and should be changed to 1.013 x 10^+5 Pa.*

**Reply:**

Thank you very much for your kind suggestion.

In accordance with the reviewer's comment, we have corrected from " 1.013×10^-5^ Pa" to "1.013×10^5^ Pa ".  (in Airflow simulation section, page 10 line 152)

Comments from Reviewer #2

(No Response)

**Reply:**

Thank you very much for reviewing our manuscript.

We look forward to hearing from you in due time regarding our submission and to respond to any further questions and comments you may have.

Sincerely,

Marie Oshima

Institute of Industrial Science, The University of Tokyo, 4-6-1 Komaba, Meguro-ku, Tokyo, 153-8505, Japan

Tel.: +81-3-5452-6206

Fax: +81-3-5452-6205

E-mail: [marie@iis.u-tokyo.ac.jp](mailto:marie@iis.u-tokyo.ac.jp)

11 April 2022
